# Multifunctional gradations of TPMS architected heat exchanger for enhancements in flow and heat exchange performances

**DOI:** 10.1038/s41598-025-04940-2

**Published:** 2025-06-06

**Authors:** Seo-Hyeon Oh, Jeong Eun Kim, Chan Hui Jang, Jungwoo Kim, Chang Yong Park, Keun Park

**Affiliations:** 1 Department of Mechanical Design and Robot Engineering, Seoul National University of Science and Technology, Seoul, Republic of Korea; 2 Department of Mechanical System Design Engineering, Seoul National University of Science and Technology, 232 Gongneung-ro, Nowon-gu, Seoul, 01811 Republic of Korea; 3 Department of Mechanical Engineering, University of Akron, Akron, OH USA

**Keywords:** Triply periodic minimal surface (TPMS), Heat exchanger, Functional gradation, Additive manufacturing, Computational fluid dynamics (CFD), Energy science and technology, Engineering

## Abstract

Heat exchangers (HXs) based on triply periodic minimal surface (TPMS) architectures have recently attracted significant interest due to their continuous and smooth shell structures with extensive surface areas. This study proposes an efficient design methodology for TPMS-based HXs by employing three gradation strategies to enhance their thermofluidic performance: (i) filtering gradation to guide hot and cold fluids through designated inlet and outlet regions with reduced flow resistance; (ii) cell-size gradation to ensure uniform flow distribution by reducing dead zones; and (iii) level-set gradation to maintain a minimum allowable wall thickness under cell-size variations. These multifunctional gradations are realized through adaptive manipulation of the signed distance fields for TPMS formulations. Computational fluid dynamics simulations were performed for various HX designs, identifying a graded design with cell sizes ranging from 6 to 10 mm as optimal for minimizing local flow stagnation. The optimized HX was fabricated via additive manufacturing and validated experimentally. Experimental results revealed a 30% improvement in heat exchange capacity with only a 0.3 kPa increase in pressure drop, resulting in a 28% enhancement in the overall heat exchange performance. These findings demonstrate that the multifunctional gradation approach enables the optimal design of TPMS-based HXs with superior thermofluidic performance and structural integrity.

**Supplementary Information:**

The online version contains supplementary material available at 10.1038/s41598-025-04940-2.

## Introduction

Additive manufacturing (AM) provides significant design flexibility compared to conventional manufacturing processes and has been utilized to fabricate functional parts with complex geometries^1^. A particularly impactful application of this flexibility lies in the development of microcellular structures, which are defined by their periodic cellular patterns, such as microlattice or triply periodic minimal surface (TPMS) structures^2^. Microlattice structures, composed of interconnected slender struts, are primarily used to develop lightweight architectures with high structural efficiency^3–6^. Beyond structural applications, microlattice structures have also been employed in functional thermofluidic components, including heat exchangers^7^, functional reactors^8^, and mold cooling modules^9^. More recently, topology optimization (TO) techniques have been integrated to further improve the thermofluidic performance of lattice-based microchannels^10–12^.

In contrast, TPMSs are surface-type cellular structures inspired by biological organisms^13^, offering continuous and smooth shell structures with extensive surface areas^14^. These TPMS structures have been utilized as biomechanical scaffolds in tissue engineering^15–17^ and as lightweight structures with high energy-absorbing capabilities^18–20^. The distinctive features of TPMS structures, including smoothly interconnected internal channels and high surface area density, have also facilitated their application in various thermofluidic systems^21^, such as heat sinks^22–24^, cooling modules or devices^25–27^, and heat exchangers^28–38^. Among these applications, heat exchangers have emerged as the most promising application, leveraging the unique ability of a TPMS to divide a three-dimensional (3D) space into two continuous domains without mutual intersection.

A heat exchanger (HX) is a component designed to facilitate heat transfer between two fluids with different temperatures. Therefore, the high area density of TPMS structures offers a distinct advantage in enhancing HX performance compared to conventional plate-based compact HXs^28^. Numerous studies have investigated the thermofluidic performance of TPMS HXs through numerical and experimental approaches. While most research has focused on unidirectional flow within TPMS channels^29–32^, bidirectional TPMS HXs have also been explored to improve heat exchange efficiency^33–35^. Recently, TPMS-based plate HXs have been developed as alternatives to traditional chevron-type plate HXs, enabling more complex 3D fluid flow and further enhancing heat transfer capabilities^36–38^.

While previous studies primarily focused on uniform TPMS cell designs, recent advancements have introduced gradual variations in cell size or wall thickness to meet specific functional requirements. This concept of functional gradation has been employed to improve the structural efficiency of TPMS structures^39–41^ and to enhance the biomedical performance of TPMS scaffolds^42–44^. More recently, gradation techniques have been applied to the design of TPMS HXs to improve heat transfer performance by altering cell types^45^ or cell sizes^46^. Notably, a meshless optimization approach was proposed to maximize heat exchange capacity by directly controlling the topology of TPMS HXs^47^. Despite these advancements, graded TPMS structures lead to non-uniform wall thicknesses, which can compromise structural integrity by introducing stress concentrations due to localized thinning.

**Fig. 1:**
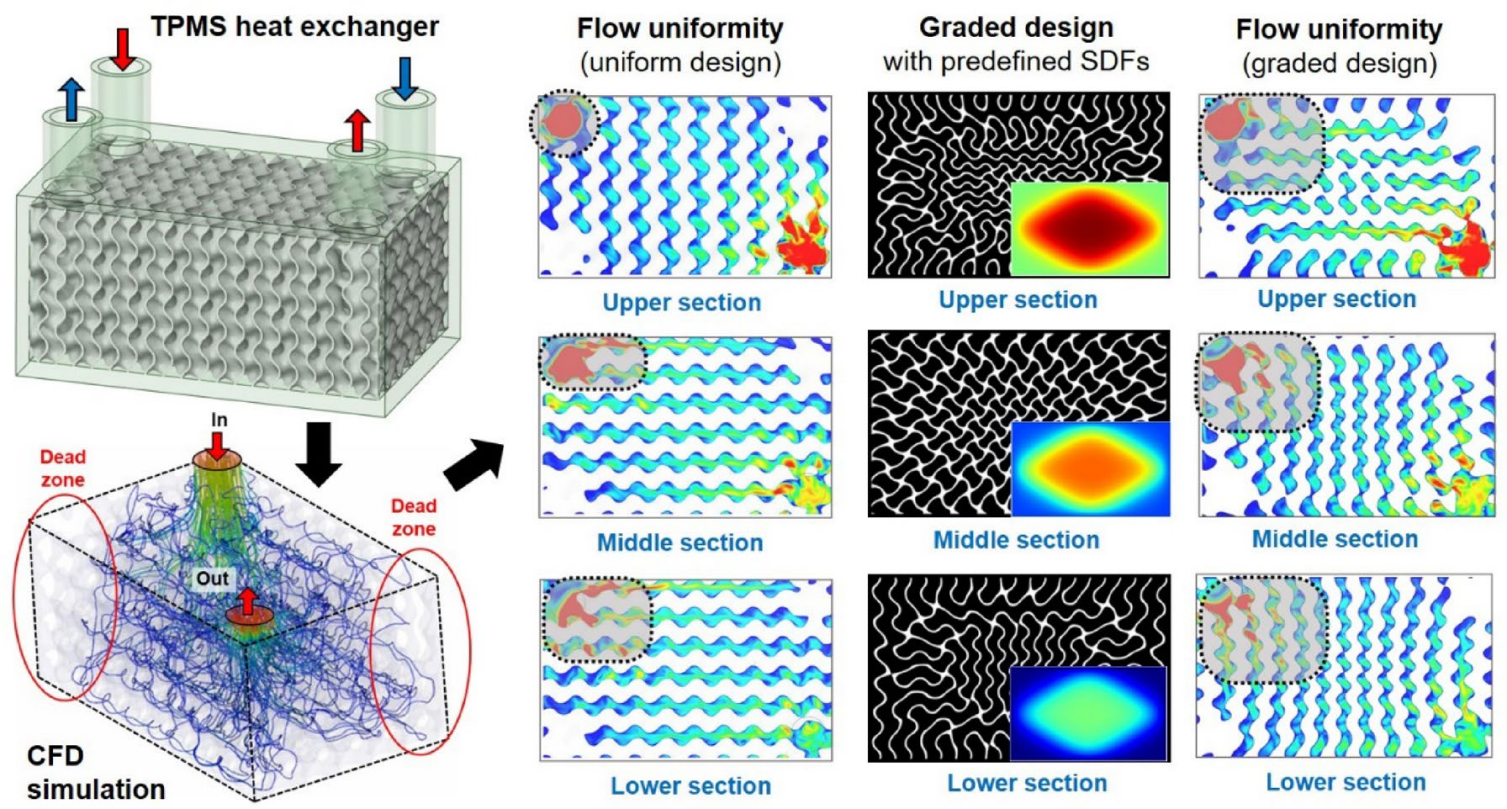
Schematic diagram of the overall design framework of the graded TPMS HXs for enhanced flow performance.

To enhance thermofluidic performance while maintaining structural integrity, this study introduces a multifunctional gradation strategy for the design of TPMS-based HXs. To this end, three types of gradations are employed to optimize the TPMS morphology. The first gradation is designed to guide hot and cold fluids through their respective inlet and outlet regions with minimized flow resistance. The second gradation gradually varies the TPMS cell size to achieve uniform flow distribution within the TPMS HX by reducing dead zones. The third gradation adjusts the level-set function for TPMS formulations to maintain a minimum allowable wall thickness, thereby ensuring structural robustness despite variations in cell size. These multifunctional gradations are implemented through extensive manipulation of TPMS formulations by adaptively modifying the signed distance field (SDF). Numerical simulations are used to analyze flow uniformity within the TPMS channels, informing the design of gradation functions for cell size and wall thickness. Figure 1 outlines the overall design framework, encompassing computational fluid dynamics (CFD) simulation to evaluate flow uniformity, SDF-based graded design, and performance evaluation for the graded TPMS HXs. The optimized TPMS HX is then fabricated via AM and experimentally validated, confirming improvements in both flow characteristics and heat exchange efficiency.

## Materials and methods

### Graded design of a TPMS structure

TPMSs are mathematically described using trigonometric combinations of sinusoidal functions. Among various TPMS types, the gyroid structure is known for its high heat transfer efficiency and superior printability, enabling robust fabrication via powder-bed-fusion (PBF) type AM techniques^37,48^. The gyroid surface can be defined mathematically by the following equation:

1 $$\phi \left(\mathbf{x}\right)=\text{sin}\left(\frac{2\pi }{l}x\right)\text{cos}\left(\frac{2\pi }{l}y\right)+\text{sin}\left(\frac{2\pi }{l}y\right)\text{cos}\left(\frac{2\pi }{l}z\right)+\text{sin}\left(\frac{2\pi }{l}z\right)\text{cos}\left(\frac{2\pi }{l}x\right)$$

where *l* denotes the size of the gyroid unit cell. Using Eq. (1), a TPMS-based shell structure can be mathematically described via level-set equations, as follows^49^:

2 $${\phi }_{sheet}\left(\mathbf{x}\right): \left\{\begin{array}{ccc}\phi \left(\mathbf{x}\right)>C, & \forall \mathbf{x}\in {\Omega }_{1}& \left(\text{Positive void}\right)\\ \left|\phi \left(\mathbf{x}\right)\right|<C,& \forall \mathbf{x}\in {\Omega }_{2} & \left(\text{Solid wall}\right) \\ \phi \left(\mathbf{x}\right)<-C,& \forall \mathbf{x}\in {\Omega }_{3}& \left(\text{Negative void}\right)\end{array}\right.$$

where *C* is the level-set constant determining the TPMS wall thickness (with *C* > 0). The entire TPMS domain is thus divided into three subdomains: positive void (${\Omega }_{1}$), solid wall (${\Omega }_{2}$), and negative void (${\Omega }_{3}$). Subsequently, the magnitude of *C* affects the wall thickness of a TPMS structure (*t*).

**Fig. 2:**
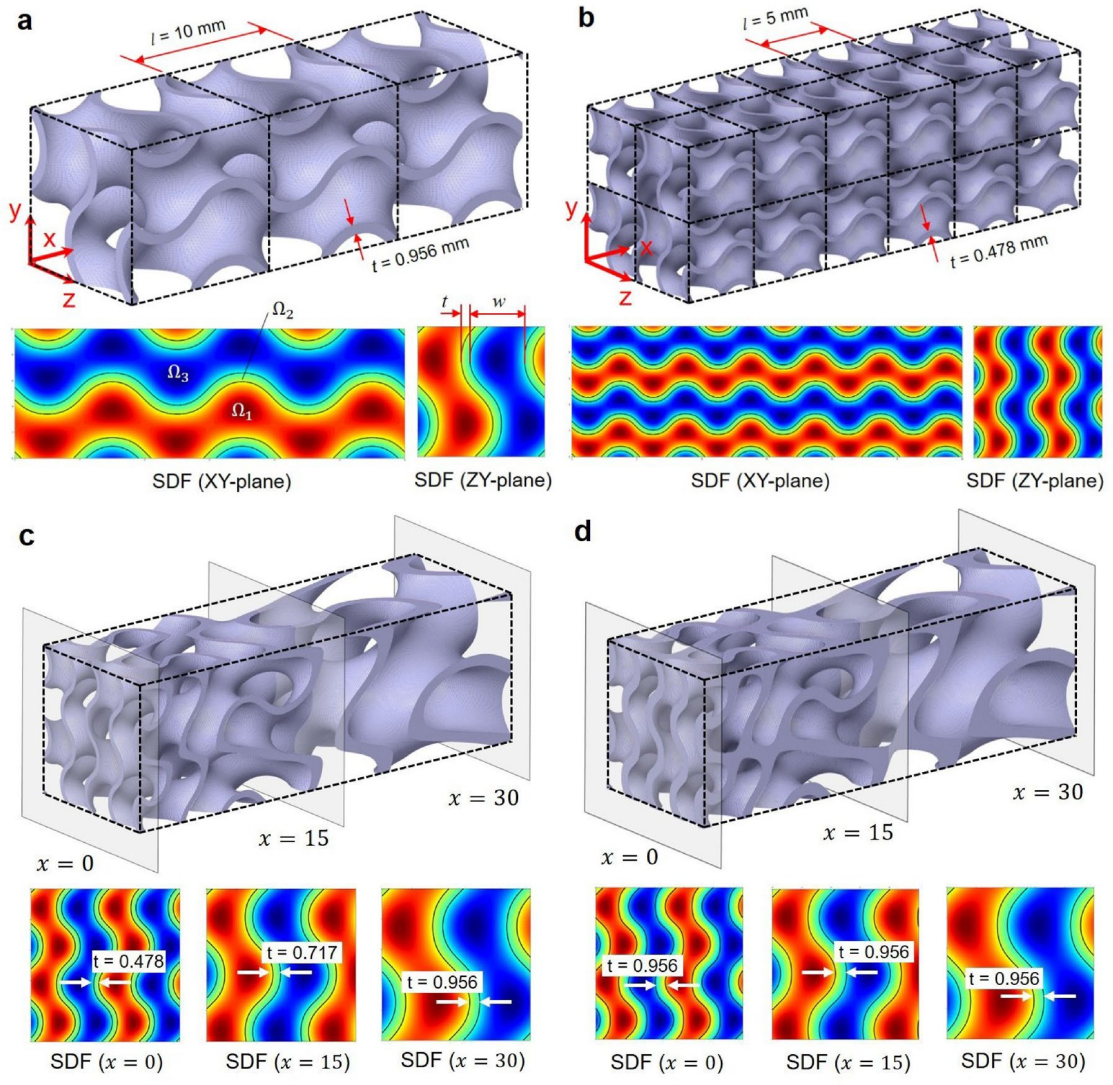
Gyroid TPMS structures and SDF distributions for different cell sizes (*l*) and level-set constants (*C*): (**a**) uniform cell size (*l* = 10 mm, *C* = 0.3), (**b**) uniform cell size (*l* = 5 mm, *C* = 0.3), (**c**) graded cell size (5 ≤ *l* ≤ 10 mm, *C* = 0.3), and (**d**) graded cell size (5 ≤ *l* ≤ 10 mm, 0.3 ≤ *C* ≤ 0.58). Here, the TPMS structure is generated in a rectangular block with a size of 30 × 10 × 10 mm.

Figure 2a illustrates a 3D TPMS structure and the relevant SDF plots, designed with *l* = 10 mm and *C* = 0.3 within a domain sized 30 × 10 × 10 mm. Under these parameters, three cells are generated along the X-direction, and the dashed lines in the figure represent the cell boundaries. In the SDF plots for the XY- and ZY-planes, the red and blue regions denote the positive (${\Omega }_{1}$) and negative (${\Omega }_{3}$) sides of the structure, corresponding to the hot and cold fluid domains, respectively. The green region indicates the solid wall of the TPMS structure (${\Omega }_{2}$), which has a thickness of 0.956 mm. Once the cell size (*l*) and wall thickness (*t*) are defined, the channel width (*w*) is calculated by the following equation.

3 $$w=\frac{1}{2}\left(l-2t\right)$$

Figure 2b presents another 3D TPMS structure and the corresponding SDF plots when *l* is reduced to 5 mm. As a result, the number of unit cells increases to 6 × 2 × 2 along the X, Y, and Z directions, respectively. Notably, the wall thickness decreases to 0.478 mm, demonstrating that the wall thickness of a TPMS structure is influenced not only by the level-set constant but also by the unit cell size. These findings also indicate that the consistent cell size results in a uniformly distributed TPMS structure with a consistent wall thickness.

In contrast, a functionally graded TPMS structure can be generated by varying the cell size based on the position vector, enabling spatial adaptation of the structure’s properties. For instance, a linearly varying cell size in the *x*-direction can be expressed as:

4 $$l\left(x\right)=\frac{({l}_{max}-{l}_{min})}{L}x+{l}_{min}$$

where *l*_*max*_ and *l*_*min*_ are the maximum and minimum cell sizes, and *L* is the total length of the TPMS domain along the X-axis. This formulation introduces a gradient in cell size, creating a TPMS structure with spatially varying properties along the X-direction.

Figure 2c depicts a TPMS structure with a linearly graded cell size, where *L*, *l*_*max*_, and *l*_*min*_ are set to 30, 10, and 5 mm, respectively. The structure demonstrates a smooth gradient in cell size across the X-direction without disconnection. The relevant cross-sectional SDF plots at varying X-positions (0, 15, and 30 mm) show that wall thickness values increase with the increase in cell size. Notably, the SDFs at *x* = 0 and *x* = 30 mm are identical to those in Fig. 2b and a, indicating that the linearly graded function effectively transitions cell size from 5 to 10 mm. However, this variation in cell size also results in a corresponding variation in wall thickness, ranging from 0.478 to 0.956 mm. Such non-uniformity in wall thickness presents a critical limitation for HX applications, as localized thinning can undermine structural integrity under fluid pressure within the TPMS channels^47^.

To address this issue and maintain a minimum allowable thickness throughout the graded TPMS structure, the level-set value must be adaptively modified in accordance with the variation in cell size. The variable level-set value is then defined by the reverse form of the cell size function, as follows:

5 $$C\left(x\right)=-\frac{({C}_{max}-{C}_{min})}{L}x+{C}_{max}$$

where *C*_*max*_ and *C*_*min*_ are the maximum and minimum level-set values. This formulation introduces a decreasing gradient in *C*(*x*) along the X-axis, counterbalancing the increasing trend in cell size described in Eq. (4). As a result, the positive effects of larger cell sizes on wall thickness are compensated by the negative impacts of reduced level-set values, maintaining a consistent wall thickness.

Figure 2d illustrates the TPMS structure with a linearly graded cell size, of which the size distribution is identical to that in Fig. 2c. Instead, the consistent wall thickness is achieved by applying the variable level-set function in Eq. (5), with *C*_*max*_ = 0.58 and *C*_*min*_ = 0.3. Figure 2d also displays the cross-sectional SDF plots at varying X-positions (0, 15, and 30 mm), confirming that the wall thickness at these locations is nearly identical. This result demonstrates that the level-set value must vary appropriately with cell size to maintain consistent wall thickness in graded TPMS structures.

### Design of a TPMS heat exchanger

**Fig. 3:**
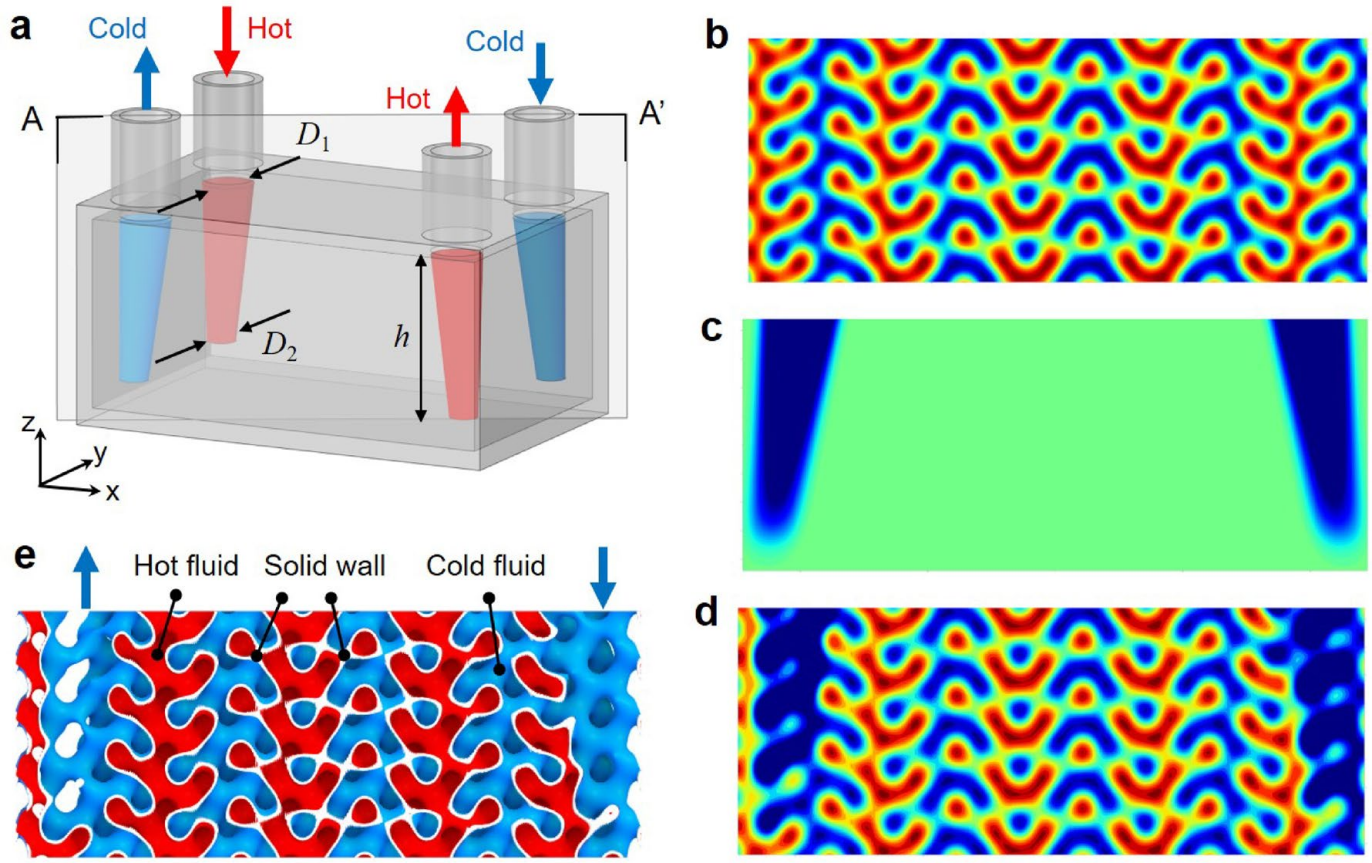
Design of a gyroid TPMS heat exchanger with tapered inlet/outlet domains: (**a**) design configuration with the filtering regions, (**b**) SDF for the original gyroid function, (**c**) SDF for the filtered inlet/outlet domains, (**d**) SDF for the filtered gyroid function, and (**e**) sectional configuration after filtering. Here, all sectional figures are prepared for the A-A’ cross-section.

The configuration of a plate-type HX with distinct inlet and outlet channels is outlined in Fig. 3a. The HX has outer dimensions of 102 × 64 × 48 mm with an external wall thickness of 2 mm, leaving a TPMS domain measuring 98 × 60 × 44 mm^50^. A gyroid-type TPMS is generated with a cell size of 10 mm. The level-set constant (*C*) is set to 0.230 to achieve a wall thickness of 0.5 mm. The HX features two vertical inlet and outlet channels designed for hot and cold fluids, as indicated by red and blue arrows in Fig. 3a, respectively. These channels regulate fluid flow into and out of the HX, and the regulation is further facilitated by circular nozzles with a diameter of 12 mm.

To ensure the selective flow of hot or cold fluids, the inlet and outlet regions of the TPMS structure are gradually changed using mathematical filtering^51^. The filtered gradation modifies the signed distance field (SDF) of the TPMS function (*ϕ*) by superimposing sigmoid functions, as follows:

6 $${\phi }^{*}\left(x,y,z\right)=\phi \left(x,y,z\right)+\sum_{i=1}^{n}\frac{{\beta }^{i}}{1+{e}^{\left.-{k}^{i}{G}^{i}(x,y,z\right)}}$$

where ${\beta }^{i}$ is the magnitude of the *i*-th sigmoid function, ${k}^{i}$ is the sigmoid coefficient, and *n* is the number of filtering functions. ${G}^{i}(x,y,z)$ is the *i*-th domain function that defines each filtered domain.

To reduce flow resistance in the inlet and outlet regions, the filtered domains were designed with tapered cylindrical shapes based on the authors’ preliminary study^52^. Four filtered domains corresponding to the inlet and outlet regions are depicted in Fig. 3a, where the hot and cold fluid regions are highlighted in red and blue arrows, respectively. These tapered cylindrical domains were designed to narrow along the flow direction, promoting the transition of fluid flow from a vertical to a horizontal direction. The mathematical expression for these filtering domains is described by:

7 $${G}^{i}\left(x,y,z\right)={\left\{x-{x}_{c}^{i}\left(z\right)\right\}}^{2}+{\left\{y-{y}_{c}^{i}\left(z\right)\right\}}^{2}-{R}^{i}{\left(z\right)}^{2}$$

where ${x}_{c}^{i}$ and ${y}_{c}^{i}$ represent the center points of the tapered cylinders, and ${R}^{i}$ represents the radius of the cylinder. These parameters linearly vary along the Z*-*axis, expressed by the following equations:

8 $${x}_{c}^{i}\left(z\right)=\frac{\left(z-{z}_{1}^{i}\right)\left({x}_{2}^{i}-{x}_{1}^{i}\right)}{\left({z}_{2}^{i}-{z}_{1}^{i}\right)}+{x}_{1}^{i}$$

9 $${y}_{c}^{i}\left(z\right)=\frac{\left(z-{z}_{1}^{i}\right)\left({y}_{2}^{i}-{y}_{1}^{i}\right)}{\left({z}_{2}^{i}-{z}_{1}^{i}\right)}+{y}_{1}^{i}$$

10 $${R}^{i}\left(z\right)=\frac{{D}_{2}^{i}}{2h}z+\frac{{D}_{1}^{i}}{2}$$

where ${D}_{1}^{i}$ and ${D}_{2}^{i}$ represent the upper and lower diameters of tapered cylinders, which were set at 12 and 6 mm, respectively. The height of the inclined region (*h*) was set to 38 mm, and the relevant center positions for four filtered domains are detailed in the supplementary information (Table S1).

Figure 3b–d provide a visual representation of the mathematical filtering process applied to the SDF plots on the A-A’ cross section. Figure 3b displays the SDF plot of the initial TPMS structure before filtering. Figure 3c presents the SDF plot of the filtering function, which features negative values (indicated by blue regions) within the filtered domains. The resulting SDF plot, after applying the filtering function, is displayed in Fig. 3d, where the filtered regions retain negative values (blue color), effectively modifying the structure to define cold fluid-exclusive areas. Consequently, the filtered regions function as cold fluid inlet and outlet channels, thereby facilitating the designated roles of the cold inlets and outlets, as shown in Fig. 3e.

### Computational fluid dynamics simulation

To explore the flow characteristics within TPMS HXs, particularly the flow uniformity through complex gyroid channels, CFD simulations were conducted using SimericsMP + ®. The standard *k-ε* model was adopted to effectively capture the turbulent behavior of fluid flow within the TPMS channels^50,53^. The simulations were based on the Reynolds-averaged Navier–Stokes (RANS) equations, with pressure–velocity coupling implemented using the steady SIMPLE scheme. The convergence for the pressure–velocity coupling was achieved by targeting residuals of less than 0.001. For the flow boundary conditions, water at 25 °C was set as the working fluid, with a flow rate of 6 L/min. The outlet boundary condition was defined by setting the gauge pressure to zero.

**Fig. 4:**
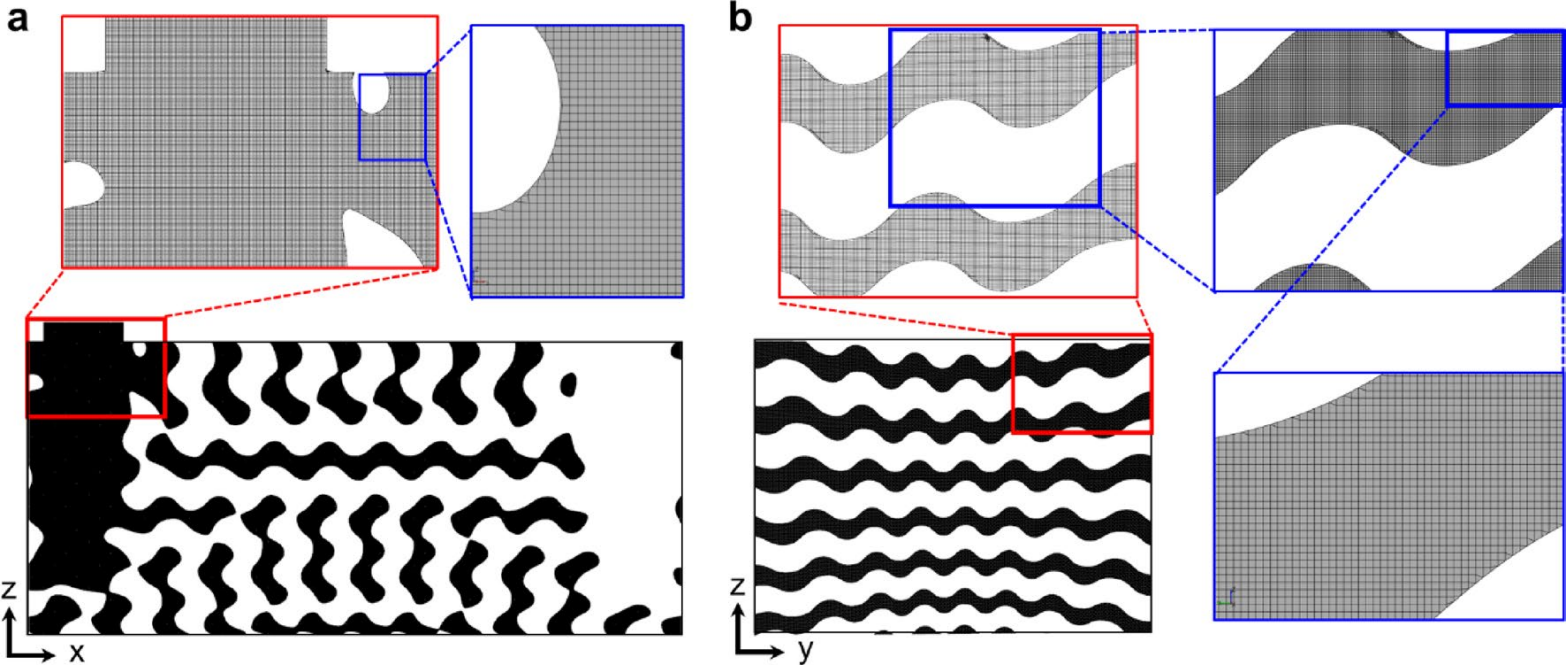
Grid structures for a TPMS fluid domain with enlarged views: (**a**) XZ-section (*y* = 52.5 mm) and (**b**) YZ-section (*x* = 49.5 mm).

The computational domain was discretized with a conformal-adaptive-binary tree mesh, which provides high efficiency and accuracy for complex TPMS geometries^54^. To accommodate the smallest channel width of 4.0 mm, a minimum grid size of 0.07 mm was applied for the fluid domain, as determined by mesh independence tests conducted in the authors’ previous study^50^. This discretization resulted in approximately 169 million cells, offering sufficient resolution to accurately capture the detailed fluid dynamics within the TPMS channels while maintaining acceptable computational cost. Figure 4a and b display the generated mesh structures for the XZ- and YZ-sections, respectively, illustrating the mesh density and its capability to capture the intricate geometrical features and flow paths within the TPMS channels.

### Additive manufacturing

The designed TPMS HXs were fabricated using a laser powder-bed-fusion (PBF) type 3D printer (CL M2 Cusing, GE Additive Inc., USA) with aluminum alloy powders (AlSi7Mg, Tekna Advanced Materials Inc., Canada). The powders, with an average particle diameter of 30 μm, yield additively manufactured parts characterized by a density of 2.67 g/cm^3^, a Brinell hardness of 70 to 85 HB, and a thermal conductivity in the range of 96–120 W/mK. Additional mechanical properties include an elastic modulus of 72.4 GPa, a yield strength ranging from 140 to 200 MPa, and an elongation at break of approximately 2% to 5%^55^.

The laser was operated at a power of 370 W with a scan speed of 1600 mm/s. Additional parameters included a layer thickness of 40 μm layer thickness, a beam spot diameter of 110 μm, and a hatching distance of 112 μm. Subsequently, the minimum feature size corresponding to these parameters was set to 200 μm. After fabrication, residual powders within the TPMS channels were removed, and the surface finish was enhanced via chemical polishing. The polishing was carried out in a solution of 85% phosphoric acid (H_3_PO_4_) and 15% copper sulfate (CuSO_4_·5H_2_O) at 130 °C for 300 s. After chemical polishing, the TPMS HX was rinsed using an ultrasonic cleaner and then subjected to a heat treatment at 280 °C for 90 min to enhance its mechanical properties.

Dimensional accuracy was evaluated by measuring the external dimensions and mass of the fabricated TPMS HXs. Surface roughness was assessed using a surface profiler (SJ-210, Mitutoyo Co., Japan). To investigate the internal structure of the fabricated TPMS HXs, a micro-CT scanner (Phoenix VTOMEX S240, Waygate Technologies, Germany) was used by analyzing X-ray computed tomography (XCT) images. The scanning resolution was set to 100 µm, allowing for the capture of the intricate internal channels within the TPMS HX. The analysis recorded a total of 1.89 billion voxels, delivering a highly detailed visualization inside the TPMS HX.

### Experiments

**Fig. 5:**
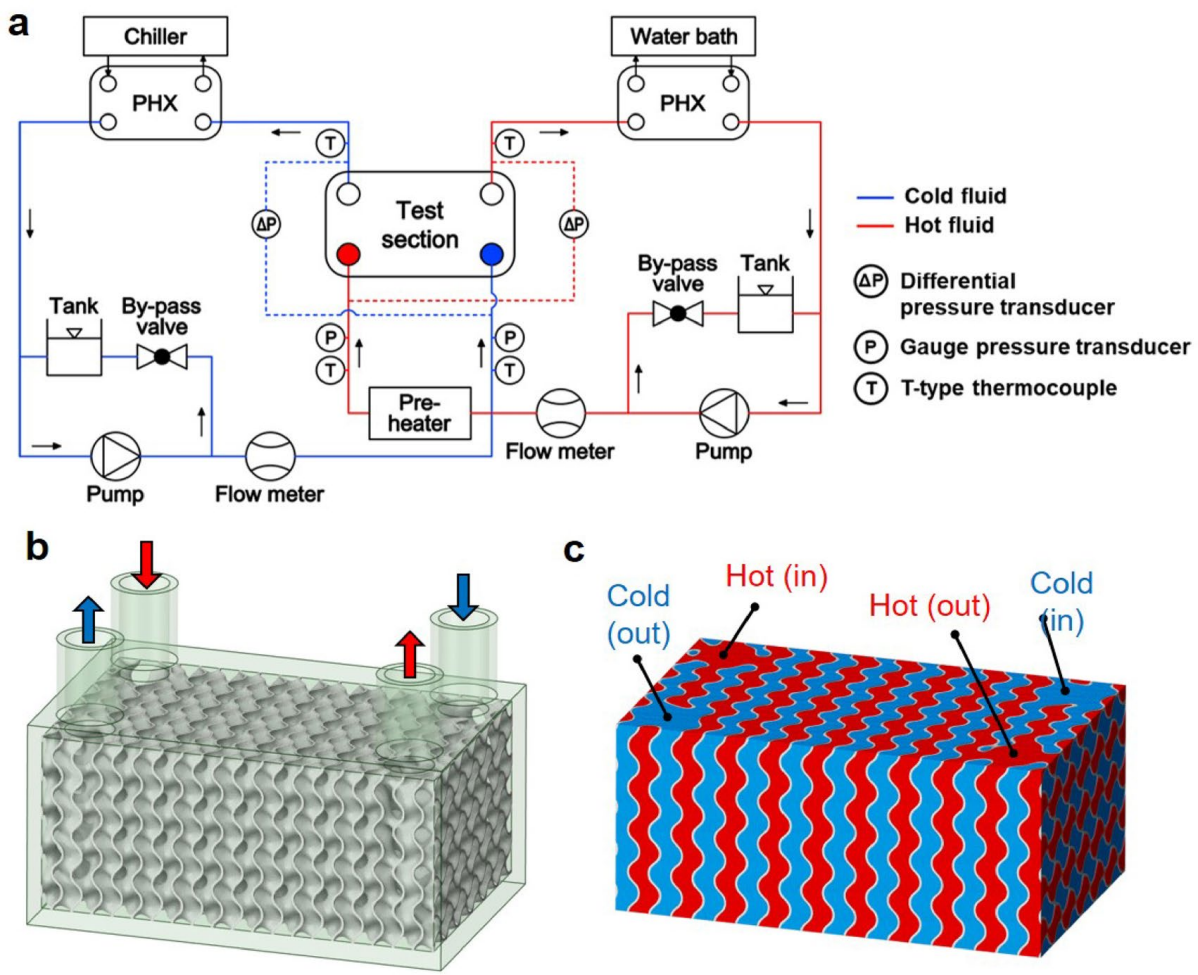
Experimental setup of TPMS HXs for the pressure drop and heat exchange tests: (**a**) schematics of the experimental setup, (**b**) designed TPMS HX structure, and (**c**) TPMS region incorporating fluid domains.

Experiments were conducted to evaluate the flow characteristics and heat exchange capabilities of TPMS HXs with different design configurations. The experimental setup, illustrated in Fig. 5a, consisted of dual circuits for hot and cold fluids. The hot fluid, a 1:1 mixture of ethylene glycol and water, was heated to a constant temperature of 25 °C using a combination of a pre-heater and a temperature-controlled water bath (RW-3040G, Jeio Tech Co. Ltd., Korea). In the cold fluid loop, pure water was chilled to 10 °C using a chiller (DX-75, Dongshin Finetek Co. Ltd., Korea). Both fluids were circulated through their respective loops by electromagnetic pumps (PM-250PMH, Wilo SE, Germany), with flow rates precisely measured using turbine flow sensors (NK-250, Korea Flowmeter Industrial Co., Korea).

To minimize the influence of environmental factors such as ambient temperature and external heat convection, the test section was insulated using thick foam insulation material. The temperatures at the inlet and outlet of each TPMS HX were measured using T-type thermocouples to ensure accurate assessment of thermal performance. The additively manufactured TPMS HXs were subjected to leakage testing, confirming no leakage under all conditions up to steady-state operation. Each steady-state condition was maintained for over one hour, ensuring stable thermal and flow behavior and enabling consistent and reliable data collection for performance assessment.

To assess the flow resistance, an additively manufactured HX was installed in the test section, with the hot fluid circulating exclusively through its inlet and outlet channels. The flow rates of the hot fluid were incrementally adjusted from 4 to 10 L/min in increments of 0.5 L/min. The pressure drop across the HX (Δ*P*) was measured using a differential pressure transducer (Model 230, Setra Systems, Inc., USA), which directly measures the pressure difference between the inlet and outlet.

To further characterize the flow resistance, the friction factor (*f*) was calculated using the following equation:

11 $$f=\frac{\Delta P\cdot {D}_{h}}{2\uprho {V}^{2}L}$$

where *L* is the flow length, *V* is the fluid velocity, and *D*_*h*_ is the hydraulic diameter of the TPMS channel. The hydraulic diameter is defined as:

12 $${D}_{h}=\frac{4\varepsilon }{{\rho }_{A}}$$

in which *ε* and *ρ*_*A*_ denote the void fraction and area density of the TPMS structure, respectively. These relationships provide a comprehensive evaluation of the pressure loss characteristics associated with the different TPMS designs.

To evaluate the heat exchange capability, both hot and cold fluids were circulated through the TPMS HXs. The flow rate of the hot fluid was maintained at a constant 8 L/min, while that of the cold fluid was adjusted between 4 and 10 L/min in increments of 0.5 L/min. Temperature and pressure measurements were taken at each fluid channel to analyze the thermofluidic performance of the additively manufactured TPMS HXs.

Figure 5b illustrates the TPMS HX configuration, which features four vertical channels designed to facilitate the cross-directional circulation of hot and cold fluids. This design ensures that each fluid flows through a separate channel, enabling efficient cross-flow interaction. Figure 5c provides a visualization of the fluid domains within the TPMS HX, where the red and blue regions represent the hot and cold fluid flow paths, respectively. The selective flow paths for each fluid in their respective inlet and outlet regions are achieved using a mathematical filtering approach, as outlined in Fig. 3, ensuring distinct and isolated flow regions for each fluid within the HX.

Using the measured temperature data, the heat exchange capacity (*Q*) values for the hot and cold fluids are calculated using the following equations:

13 $${Q}_{h}={\dot{m}}_{h}\cdot {C}_{h}\cdot \left({T}_{h}^{i}-{T}_{h}^{o}\right)$$

14 $${Q}_{c}={\dot{m}}_{c}\cdot {C}_{c}\cdot \left({T}_{c}^{o}-{T}_{c}^{i}\right)$$

where ${\dot{m}}_{h}$ and ${\dot{m}}_{c}$ are the mass flow rates of the hot and cold fluids, respectively, and ${C}_{h}$ and ${C}_{c}$ are the specific heat of the hot and cold fluids. The temperatures of the hot and cold fluids are represented by ${T}_{h}$ and ${T}_{c}$, with the superscripts *i* and *o* indicating the inlet and outlet conditions.

The overall heat transfer coefficient (*U*) was calculated using the following equations:

15 $$U=\frac{Q}{\Delta {T}_{LM}\cdot A}$$

where *A* is the heat transfer area and *ΔT*_*LM*_ is the logarithmic mean temperature difference based on the temperature variations of the hot and cold fluids, as follows^56^:

16 $$\Delta {T}_{LM}=\frac{\left({T}_{h}^{i}-{T}_{c}^{i}\right)-\left({T}_{h}^{o}-{T}_{c}^{o}\right)}{\text{ln}\{\left({T}_{h}^{i}-{T}_{c}^{i}\right)/({T}_{h}^{o}-{T}_{c}^{o})\} }.$$

To comprehensively evaluate the performance of the TPMS HXs, the Colburn *j*-factor was calculated, defined by:

17 $$j=\frac{Nu}{Re\cdot {Pr}^{1/3}}$$

where the dimensionless quantities *Nu*, *Re*, and *Pr* represent the Nusselt, Reynolds, and Prandtl numbers, respectively. These equations facilitated the assessment of heat transfer performance and efficiency for TPMS HXs with various graded designs under diverse operating conditions.

### Uncertainty analysis

An uncertainty analysis was performed to validate the reliability of the experimental results. Fluid temperatures at the inlets and outlets of the test section were measured using T-type thermocouples with an accuracy of ± 0.2 °C, ensuring precise monitoring of temperature variations essential for assessing heat exchange performance. Pressure drops across the TPMS test section were accurately measured using differential pressure transducers (Model 230, Setra Systems, Inc., USA) with an accuracy of ± 0.25% FS (± 0.1724 kPa), providing dependable data on flow resistance. Additionally, a digital flowmeter (NK-250, Korea Flowmeter Industrial Co., Korea) with an accuracy of ± 0.5% FS (0.09 L/min) was used to measure the volumetric flow rates of both hot and cold fluids, ensuring consistent and reliable flow condition data for evaluating the heat exchanger’s performance.

The uncertainties associated with the overall performance indices were calculated by propagating errors from measurements of temperature, pressure, and flow rate. These propagated uncertainties, as detailed in Table 1, underscore the reliability of the experimental data and its influence on the derived thermo-fluidic performance metrics. This analysis ensures confidence in the accuracy and validity of the evaluated flow resistance and heat exchanger performance.

**Table 1:** Measurement uncertainties of the overall performance indices.

Performance index	Uniform	Graded
Pressure drop [Δ*P*]	± 8.54%	± 7.03%
Friction factor [*f*]	± 8.91%	± 7.58%
Nusselt number [Nu]	± 9.60%	± 7.88%
Heat exchange capacity [*Q*]	± 5.94%	± 4.61%
Overall heat transfer coefficient [*U*]	± 6.19%	± 4.98%

## Results and discussion

### Design of graded TPMS heat exchangers

In this section, three gradation strategies introduced in “Graded design of a TPMS structure” and “Design of a TPMS heat exchanger” sections are further investigated to enhance the performance of TPMS-based HXs. The first strategy, termed *filtered gradation*, introduces tapered cylindrical domains to guide fluid flow and is validated through CFD simulations under varying cell sizes. Based on the simulation findings, the second gradation involves spatial variation of the cell size to improve flow uniformity within the TPMS domain. To complement this, a third gradation is applied to preserve consistent wall thickness despite the introduced cell-size variation, thereby maintaining structural integrity. Detailed descriptions of each gradation strategy are provided in the following subsections.

#### Effect of the cell size

**Fig. 6:**
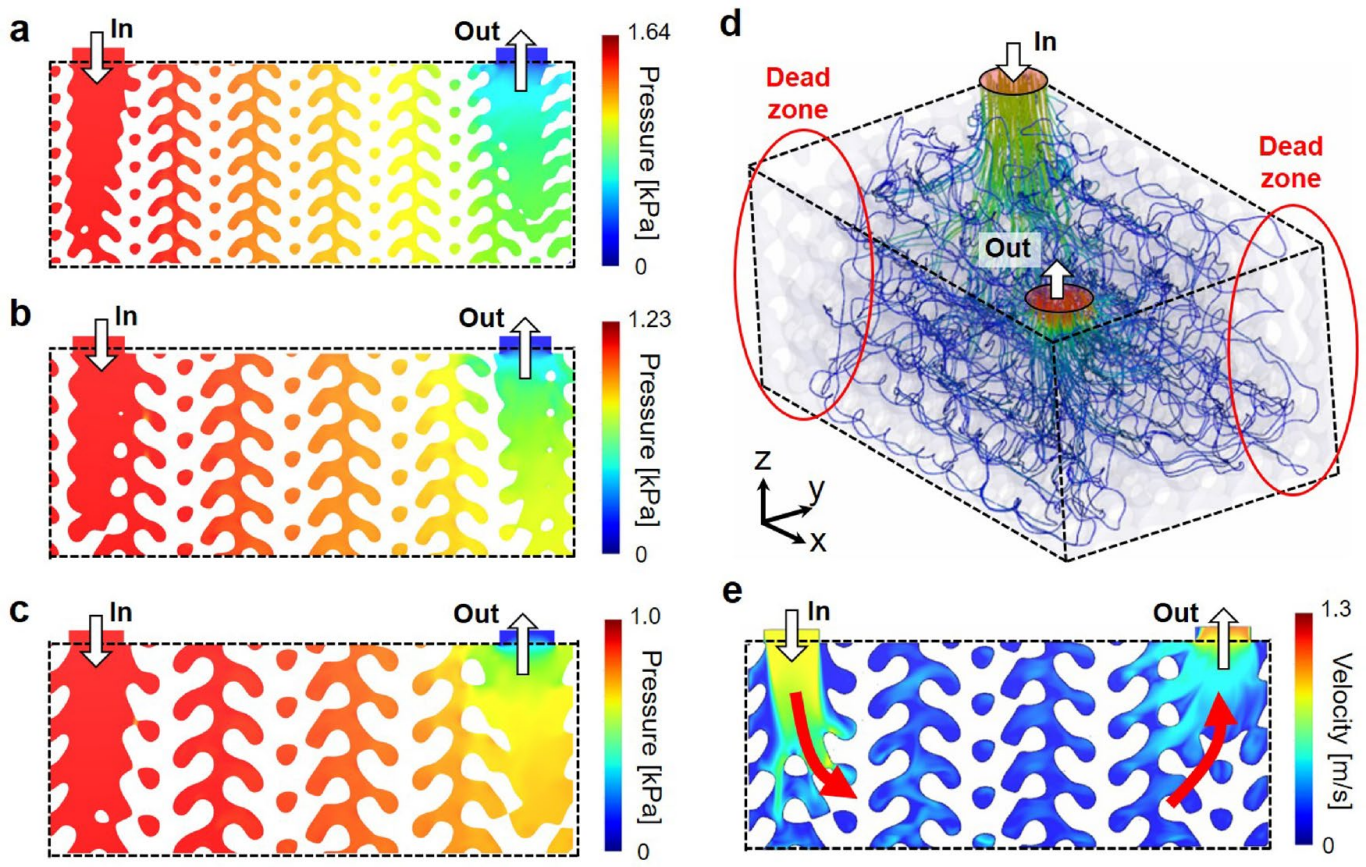
CFD simulation results for the uniform HX design with different cell sizes: (**a**) sectional pressure distribution (*l* = 6 mm), (**b**) sectional pressure distribution (*l* = 8 mm), (**c**) sectional pressure distribution (*l* = 10 mm), (**d**) 3D streamline with velocity distribution (*l* = 10 mm), and (**e**) sectional velocity distribution (*l* = 10 mm).

To investigate the effect of cell size on the flow characteristics, CFD simulations were conducted for uniform TPMS designs with different cell sizes (*l* = 6, 8, and 10 mm). In all cases, the TPMS wall thickness was maintained at 0.5 mm by adjusting the level-set constants. Figure 6a− c illustrate the pressure distributions of three design cases across the diagonal cross-sections. The pressure drop (Δ*P*) increases with decreasing cell size, showing a 63.1% increase from *l* = 10 mm (1.004 kPa) to *l* = 6 mm (1.632 kPa).

To further interpret these results, geometric properties of the TPMS structures, including the porosity, area density, and hydraulic diameter, are summarized in Table 2. As the cell size increases, the porosity rises from 73.9% to 85.4% while the area density decreases from 515.4 to 287.8 m^2^/m^3^. This trend suggests improved flow characteristics due to reduced flow resistance, but potentially reduced heat transfer performance resulting from a decrease in available surface area. Notably, the 10 mm cell size yields the highest hydraulic diameter of 5.95 mm, further contributing to improved flow conditions.

**Table 2:** Comparison of designed TPMS features and the relevant hydraulic and structural performance.

*l* (mm)	Porosity (%)	Area density (m^2^/m^3^)	Hydraulic diameter (mm)	Δ*P* (kPa)	*σ*_max_ (MPa)
6	73.9	515.37	3.03	1.632	56.75
8	81.5	386.54	4.31	1.237	44.31
10	85.4	287.76	5.95	1.004	35.79

Another critical consideration in TPMS HX design is structural safety, as differences in porosity and internal pressure can influence stress distributions within the TPMS walls. To address this, structural finite element analyses (FEAs) were conducted for the three TPMS configurations using ANSYS Workbench (ANSYS Inc., USA) under an internal pressure of 5 MPa. The resulting maximum equivalent stress (*σ*_max_) values are listed in Table 2, indicating that smaller cell sizes yield higher stress levels. Although all *σ*_max_ values (35.79–56.75 MPa) remain below the yield strength (140–200 MPa), these stress levels should be maintained as low as possible to mitigate potential thermal fatigue during repeated heat exchanger operation.

Based on the simulation results, the optimal cell size was determined to be 10 mm. Although increasing the cell size beyond this value could further reduce flow resistance, it may compromise manufacturability due to the formation of overhang features, which are prone to failure during the AM process. Instead, this design was further explored through CFD simulation to enhance flow uniformity within the TPMS channels.

Figure 6d illustrates the 3D streamlines within the TPMS HX, with the flow velocity magnitude represented by the color scale. It can be seen that the injected fluid flows diagonally from the inlet to the outlet, resulting in dead zones due to flow stagnation in the two other corner regions. These dead zones deteriorate flow uniformity within the TPMS HX and thus need to be minimized to enhance the heat exchange performance. Figure 6e presents the sectional velocity distribution along the diagonal cross-section, revealing that the injected fluid flows downward and horizontally, an effect attributed to the tapered inlet design. A similar trend of horizontal and upward flow is observed near the outlet region. Although the introduction of horizontal flow improves flow uniformity^52^, it is predominantly developed in the lower part of the domain, as highlighted in Fig. 6e. Therefore, additional TPMS gradation is required to promote horizontal flow in the upper region and further improve uniformity.

#### Gradation for flow uniformity

To enhance flow uniformity, the TPMS HX is redesigned using graded cell sizing, where the cell size is reduced in high-velocity regions. In the graded design, the cell size varies gradually in 3D space and is defined by the following equation:

18 $$l\left(x,y,z\right)={\lambda }_{1}{l}_{1}\left(z\right)+{{\lambda }_{2}l}_{2}\left(x,y\right)$$

where ${l}_{1}\left(z\right)$ and ${l}_{2}\left(x,y\right)$ represent the out-of-plane and in-plane gradation functions, respectively, while ${\lambda }_{1}$ and ${\lambda }_{2}$ are the associated weight factors.

**Fig. 7:**
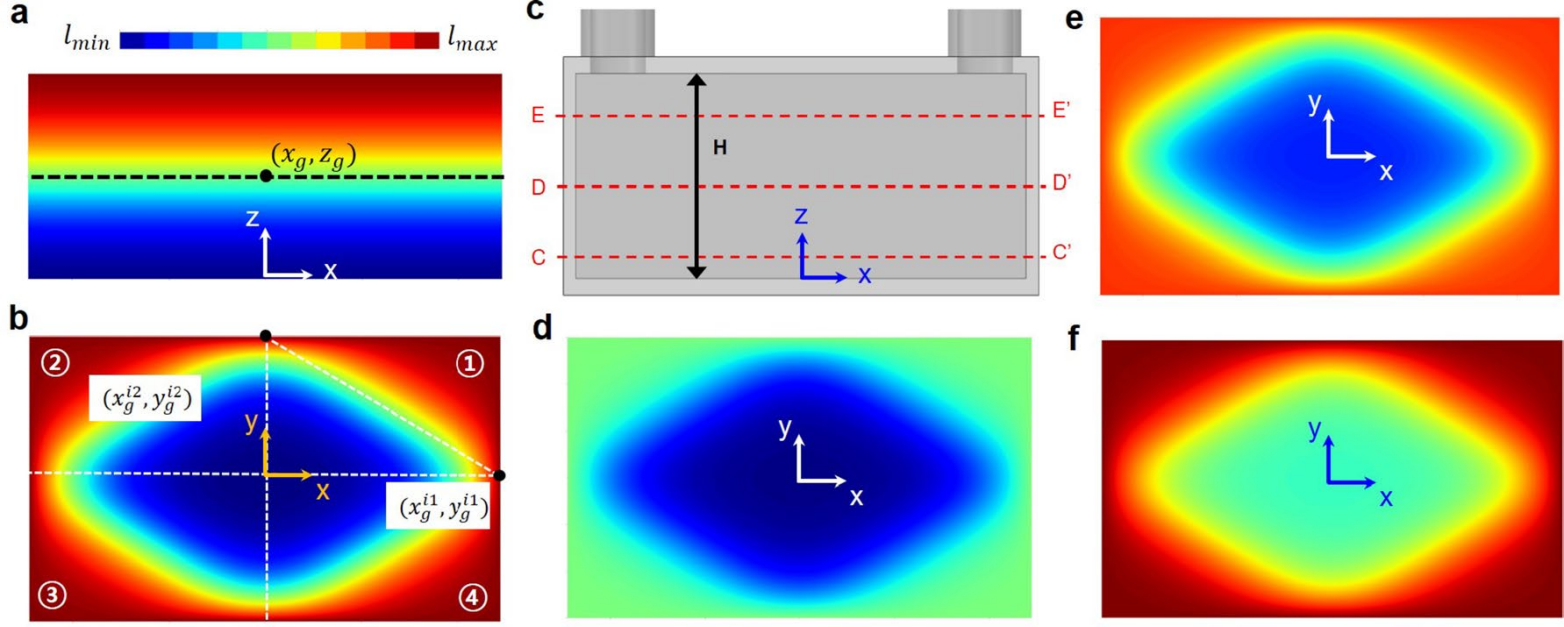
Gradation strategy within the TPMS HX: (**a**) out-of-plane distribution (XZ-plane) and (**b**) in-plane distribution (XY-plane). Distributions of cell size at different cross-sections: (**c**) configuration of cross-sections, (**d**) C–C’ cross-section (*z/H* = 0.05), (**e**) D-D’ cross-section (*z/H* = 0.45), and (**f**) E-E’ cross-section (*z/H* = 0.85).

The out-of-plane gradation function is defined to decrease the cell size along the downward direction as a form of a sigmoid function:

19 $${l}_{1}\left(z\right)=\frac{{\beta }_{1}}{1+{e}^{-{k}_{1}(z-{z}_{g})}}$$

where ${\beta }_{1}$ represents the magnitude of the filtering function, scaled to normalize the function range to match the specified minimum and maximum cell sizes. The parameter ${k}_{1}$ is a sigmoid coefficient that controls the transition rate, while ${z}_{g}$ denotes the Z-position of the reference point that defines the center of the gradation^51^. These parameters are empirically chosen based on the intended location and rate of the transition in cell size. Figure 7a illustrates the resulting cell size distributions on the XZ-section, corresponding to (${l}_{1}\left(z\right)$), where the minimum and maximum cell sizes were set to 8 and 10 mm, respectively. In this configuration, ${k}_{1}$ and ${z}_{g}$ were assigned values of 0.15 and 0, respectively, to reduce the cell size below the bottom of the tapered cylindrical domains.

In contrast, the in-plane gradation function is defined to increase cell size in the four corner regions while reducing it in the central region. For this purpose, the in-plane domain is divided into four quadrants, as depicted in Fig. 7b. The cell size function for each quadrant is constructed to exhibit a diagonal gradient. For instance, the size function for the first quadrant (${l}_{2}^{1}(x,y)$) is expressed as:

20 $${l}_{2}^{1}(x,y)=\frac{{\beta }_{2}}{1+{e}^{-{k}_{2}\left[\left({y}_{g}^{i1}-{y}_{g}^{i2}\right)x-\left({x}_{g}^{i1}-{x}_{g}^{i2}\right)y+\left({x}_{g}^{i1}{y}_{g}^{i2}-{x}_{g}^{i2}{y}_{g}^{i1}\right)\right]}}$$

where ${k}_{2}$ represents the sigmoid coefficient, ${\beta }_{2}$ is the scale coefficient for normalizing, and $\left({x}_{g}^{i1},{y}_{g}^{i1}\right)$ and $({x}_{g}^{i2},{y}_{g}^{i2})$ are control points within the first quadrant. To construct the overall in-plane size function across the XY-plane, the size functions of all four quadrants are superposed, as given by the following equation.

21 $${l}_{2}\left(x,y\right)=\sum_{i=1}^{4}{l}_{2}^{i}(x,y).$$

Figure 7b illustrates the in-plane cell size distribution on the XY-section (${l}_{2}\left(x,y\right)$), with minimum and maximum cell sizes set to 8 and 10 mm, respectively. In this configuration, two control points for the first quadrant are located at (49, 0) and (0, 30), positioned near the midpoints of the two outer edges to effectively introduce cell size differentiation in the corner regions. The sigmoid coefficient (${k}_{2}$) was set to 0.004 to ensure a gradual change in the cell size distribution, specifically increasing cell sizes in the dead zones. The same parameter settings used for the first quadrant were symmetrically applied to the remaining three quadrants to maintain overall geometric consistency.

The combined cell size distribution is derived by superimposing the out-of-plane and in-plane distribution functions, as defined in Eq. (18). Figure 7d−f present the resultant cell size distributions across three different cross-sections (sections C–C’, D-D’, and E-E’), of which section definitions are depicted in Fig. 7c, demonstrating a smoothly graded configuration along both in-plane and out-of-plane directions. This gradation is expected to promote better flow uniformity by achieving a balanced gradation throughout the structure.

#### Gradation for uniform wall thickness

**Fig. 8:**
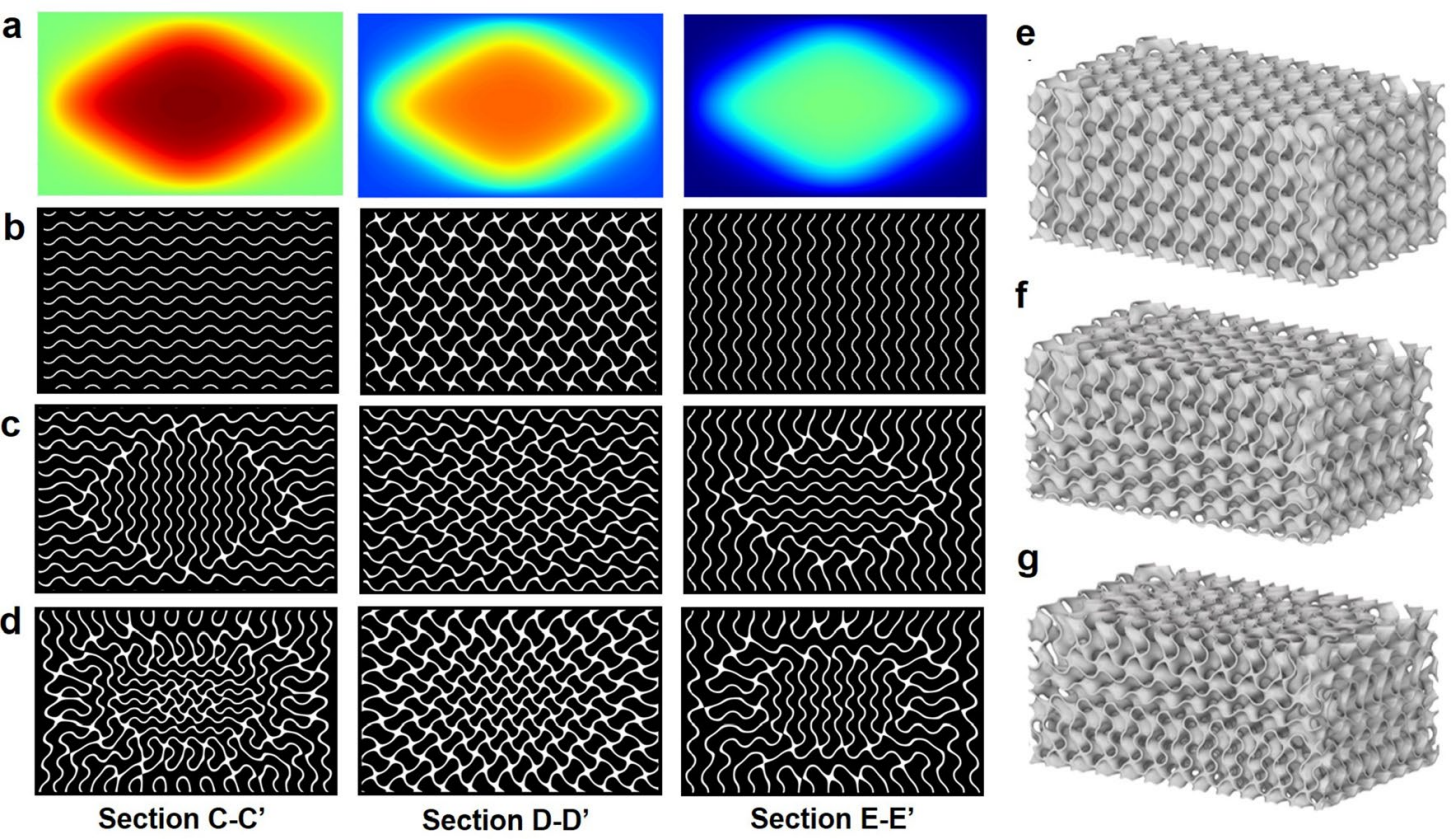
Multifunctional gradation combining the cell-sizing and level-set gradations: (**a**) distributions of the level-set functions, sectional images for three cross-sections: (**b**) uniform TPMS (*l* = 10 mm), (**c**) graded TPMS (*l*_*min*_ = 8 mm), and (**d**) graded TPMS (*l*_*min*_ = 6 mm), and designed TPMS structures with different gradations: (**e**) uniform TPMS (*l* = 10 mm), (**f**) graded TPMS (*l*_*min*_ = 8 mm), and (**g**) graded TPMS (*l*_*min*_ = 6 mm).

To incorporate the graded size distribution into the TPMS design, the level-set values must be adjusted to maintain consistent wall thickness. As outlined in “Graded design of a TPMS structure” section, the level-set function described in Eq. (5) is directly related to the cell size function given in Eq. (4). Using these relationships, the distributions of the level-set function were determined as depicted in Fig. 8a. These distributions correspond to the cell size variations shown in Fig. 7, demonstrating that the level-set function exhibits an inverse distribution compared to the cell size function across the three cross-sections.

These graded sizes and level-set functions were applied to two graded designs with minimum cell sizes (*l*_*min*_) of 8 mm and 6 mm. The maximum cell size was set at 10 mm, identical to the uniform design. To maintain a wall thickness of 0.5 mm, the level-set values were adjusted, and the ranges for these values are detailed in Table 3. While the uniform design utilizes a constant level-set value of 0.230, the graded designs require higher level-set values due to reduced cell size. Specifically, the maximum level-set values (*C*_*max*_) reached 0.282 for the *l*_*min*_ = 8 mm case and 0.375 for the *l*_*min*_ = 6 mm case.

**Table 3:** Comparison of level-set values and the geometric properties of TPMS HXs with different gradations.

TPMS design	Level-set values		Porosity (%)	Area density	Hydraulic diameter (mm)
	*C*_*min*_	*C*_*max*_		(m^2^/m^3^)	
Uniform (*l* = 10 mm)	0.230	0.230	85.4	287.76	5.95
Graded (*l*_*min*_ = 8 mm)	0.230	0.282	81.4	320.68	5.10
Graded (*l*_*min*_ = 6 mm)	0.230	0.375	80.6	350.72	4.59

The resulting TPMS configurations are illustrated through sectional images in Fig. 8b–d. In these images, the white regions represent the TPMS walls, and the black regions denote the voids. Figure 8b illustrates the uniform design, characterized by consistently repeated patterns in each cross-section. Conversely, Fig. 8c and d reveal morphological changes in the TPMS walls reflecting the graded designs. Despite these variations, the wall thickness remains consistently maintained at 0.5 mm across all designs, except at cross-points. These findings confirm that the proposed level-set function effectively controls wall thickness, even in complex graded TPMS designs.

Figure 8e–g illustrate the generated TPMS structures with different size gradations. The uniform design in Fig. 8e exhibits consistently repeated patterns throughout, whereas the graded designs in Fig. 8f and g reveal noticeable morphological changes in the arrangement of TPMS cells. Table 3 provides detailed geometric properties for the three designs, including porosity, area density, and hydraulic diameter. These results indicate that both graded designs maintain high porosity levels (> 80%) and area densities exceeding 320 m^2^/m^3^, which are beneficial for reducing flow resistance and enhancing heat exchange performance. Notably, the graded design with a smaller minimum cell size (e.g., 6 mm) leads to a reduced hydraulic diameter and an increased area density. This outcome suggests a trade-off because a reduced hydraulic diameter implies lower flow performance, while an increased area density enhances heat transfer capability. These competing effects will be further examined through CFD simulation in the subsequent section to assess their impact on flow uniformity.

### Flow characteristics inside graded TPMS designs

**Fig. 9:**
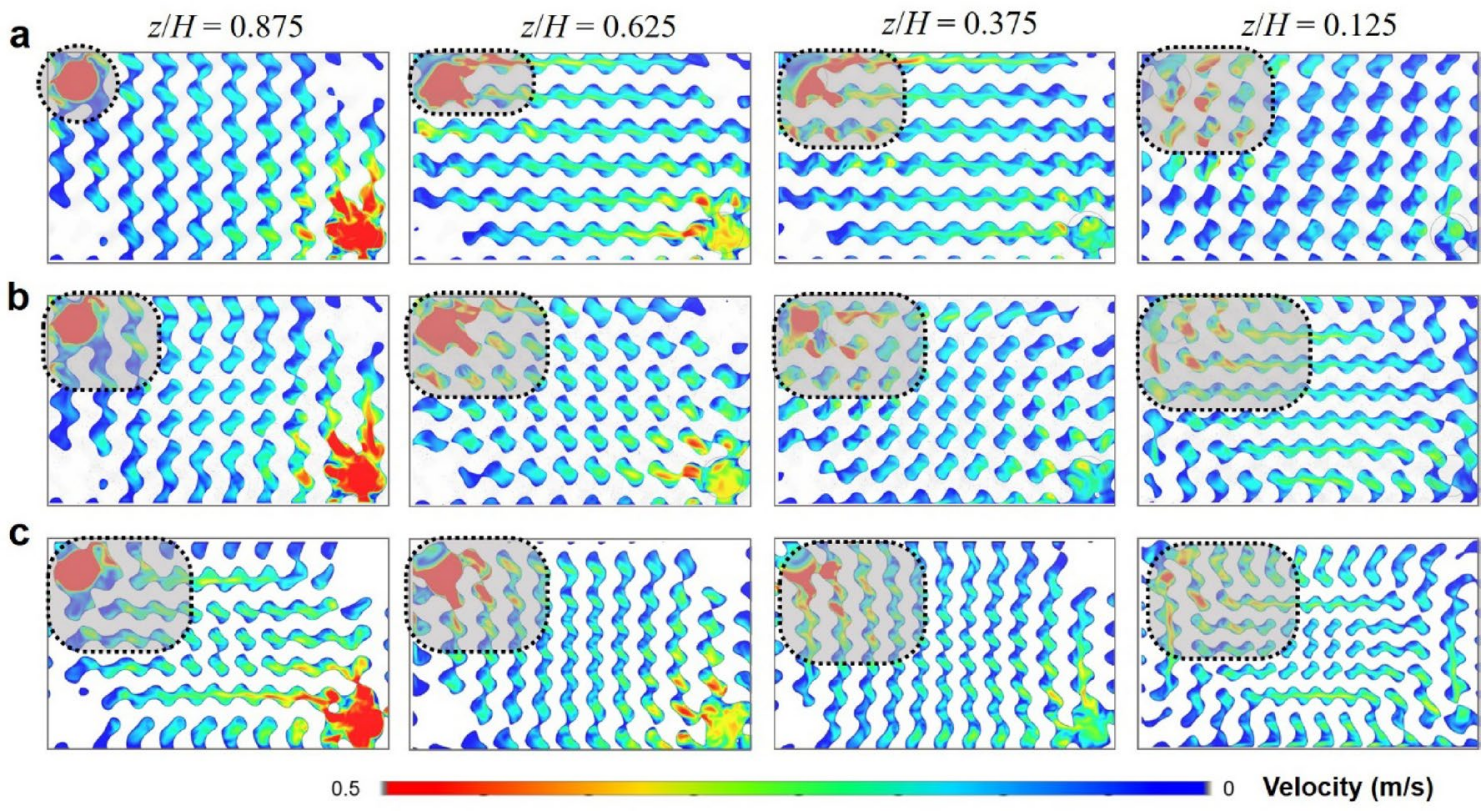
Flow velocity distributions inside the TPMS HX on different cross sections (unit: m/s): (**a**) uniform TPMS (*l* = 10 mm), (**b**) graded TPMS (*l*_*min*_ = 8 mm), and (**c**) graded TPMS (*l*_*min*_ = 6 mm).

CFD simulations were additionally performed to evaluate the flow characteristics of two graded TPMS designs with different minimum cell sizes (*l*_*min*_ = 8 and 6 mm), compared to those of the uniform TPMS with constant cell size (*l* = 10 mm). Figure 9 presents the sectional velocity distributions for these designs, with four XY-sections at different vertical positions (i.e., *z/H* = 0.875, 0.625, 0.375, and 0.125) selected for comparison.

Figure 9a shows the velocity distributions within the uniform TPMS at different vertical positions, wherein the high-velocity zones are highlighted. The high-velocity zone is initially concentrated near the inlet region at *z/H* = 0.875, and progressively expands as the flow moves downward. This trend indicates a lack of flow uniformity, as evidenced by the concentration of high velocities in the lower regions, consistent with the findings in Fig. 6e.

In contrast, the graded TPMS designs exhibit significantly improved flow uniformity. Figure 9b shows the velocity distribution for the first graded design (*l*_*min*_ = 8 mm), where high-velocity zones are more evenly distributed compared to the uniform TPMS. The second graded design (*l*_*min*_ = 6 mm), shown in Fig. 9c, further enhances flow uniformity. This improvement in flow uniformity can be attributed to the variations in channel width introduced by the graded configurations. The in-plane gradation reduces cell size and channel width in the central area, directing flow to corner regions with relatively wider channels, which alleviates stagnation in the corner regions. Simultaneously, the out-of-plane gradation decreases cell size and channel width along the downward direction, maintaining a consistent distribution of high-velocity zones across different vertical locations.

**Fig. 10:**
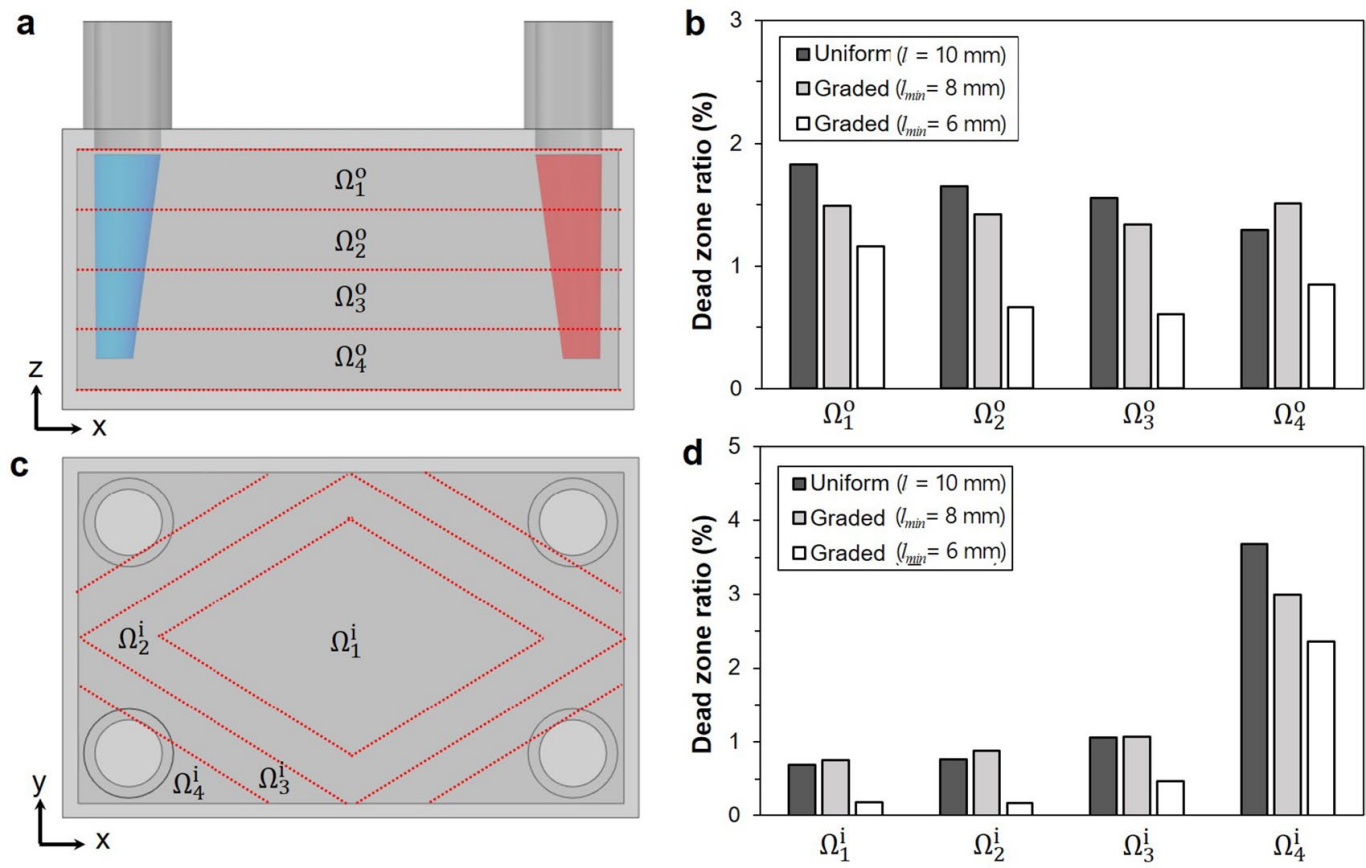
Comparison of the dead-zone ratio for three TPMS designs: (**a**) definition of out-of-plane subdomains, (**b**) comparison of out-of-plane dead-zone ratio, (**c**) definition of in-plane subdomains, (**d**) comparison of in-plane dead-zone ratio.

To facilitate quantitative comparison, the distributions of dead flow zones were analyzed by segmenting the TPMS domain into finite subdomains. Figure 10a depicts the division of the out-of-plane domain into four subdomains (${\Omega }_{1}^{o}, {\Omega }_{2}^{o}, {\Omega }_{3}^{o}$, and ${\Omega }_{4}^{o}$), with the corresponding dead-zone ratios for each TPMS design presented in Fig. 10b. Here, dead zones were defined as regions with negligible flow velocity, less than 0.001 m/s. The uniform TPMS design exhibits the highest dead-zone ratio, ranging from 1.29 to 1.83%. In contrast, the dead-zone ratios of the graded TPMS designs are reduced significantly, achieving values between 0.61% and 1.16% when *l*_*min*_ was set to 6 mm.

Similarly, the in-plane domain is divided into four subdomains (${\Omega }_{1}^{i}, {\Omega }_{2}^{i}, {\Omega }_{3}^{i}$, and ${\Omega }_{4}^{i}$), as shown in Fig. 10c. The resulting dead-zone ratios for each subdomain are illustrated in Fig. 10d, revealing the highest values in the outer subdomain (${\Omega }_{4}^{i}$). In this subdomain, the uniform TPMS design exhibited a dead-zone ratio of 3.68%, which was reduced to 2.35% in the graded design with a minimum cell size of 6 mm.

### Experimental validation

**Fig. 11:**
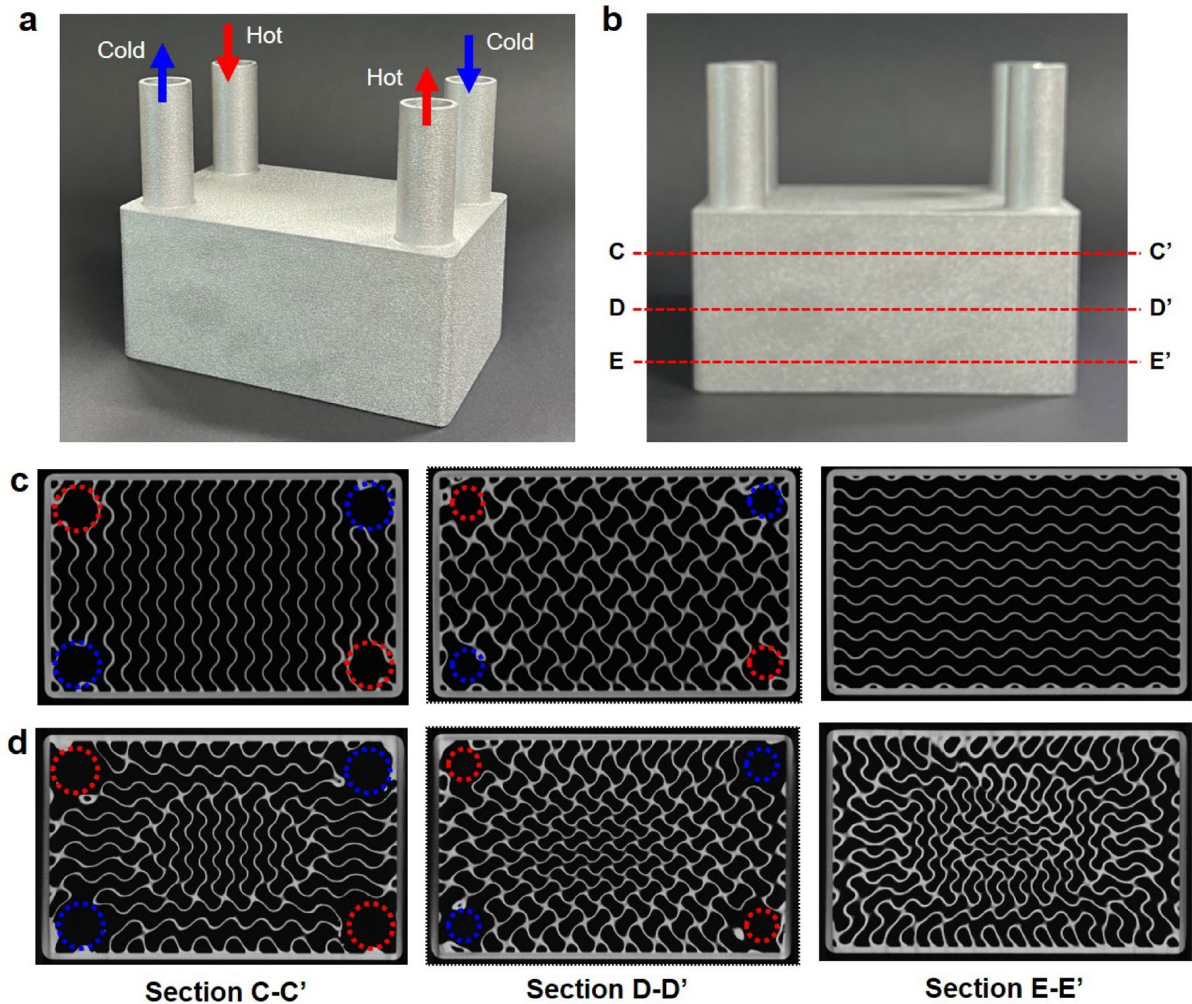
Additively manufactured TPMS HXs (*l* = 10 mm): (**a**) isometric view and (**b**) side view with cross-section definitions. XCT images of the fabricated TPMS HXs at different cross-sections: (**c**) uniform TPMS (*l* = 10 mm) and (**d**) graded TPMS (*l*_*min*_ = 6 mm).

Based on the CFD simulation results, the graded TPMS HX design with cell sizes ranging from 6 to 10 mm was selected and additively manufactured for experimental validation. For comparative analysis, a uniform TPMS HX was also fabricated, as shown in Fig. 11a and b with sectional definitions. Detailed comparisons of the outer dimensions and mass with their design specifications are provided in the supplementary information (Table S2), confirming the high dimensional accuracy achieved through the AM process. Surface roughness measurements indicated an initial roughness of 15.90 μm, which was significantly reduced to 6.63 μm following chemical polishing.

Figure 11c and d present XCT images of the two TPMS HXs at various cross-sections, confirming that the TPMS walls were fabricated without defects. Furthermore, these sectional images closely match the designed structures: Fig. 8b for the uniform design and Fig. 8d for the graded design. Notably, the four corner regions in sections C–C’ and D-D’ contain circular holes that gradually diminish in size, as highlighted by dashed circles. These features correspond to the inlet and outlet configurations for the hot and cold fluids based on the filtered gradation, as illustrated in Fig. 3. In contrast, section E-E’ does not exhibit such holes, as this region remains unaffected by the filtering gradation. Wall thickness measurements obtained from the XCT images were 0.526 ± 0.04 mm for the uniform design and 0.533 ± 0.05 mm for the graded design, corresponding to deviations of approximately 5.5% and 6.6%, respectively, from the designed thickness of 0.5 mm.

Experiments were then conducted using these additively manufactured TPMS HXs, following the experimental setup described in “Experiments” section, Across the flow rate range of 4 to 10 L/min, an energy balance evaluation using Eqs. (13) and (14) revealed that 92.3% of the experimental data exhibited a discrepancy of less than ± 5% between the heat transfer rates calculated from the hot and cold fluid loops. These results demonstrate the consistency and reliability of the experimental setup in assessing the thermofluidic performance of the TPMS HXs.

**Fig. 12:**
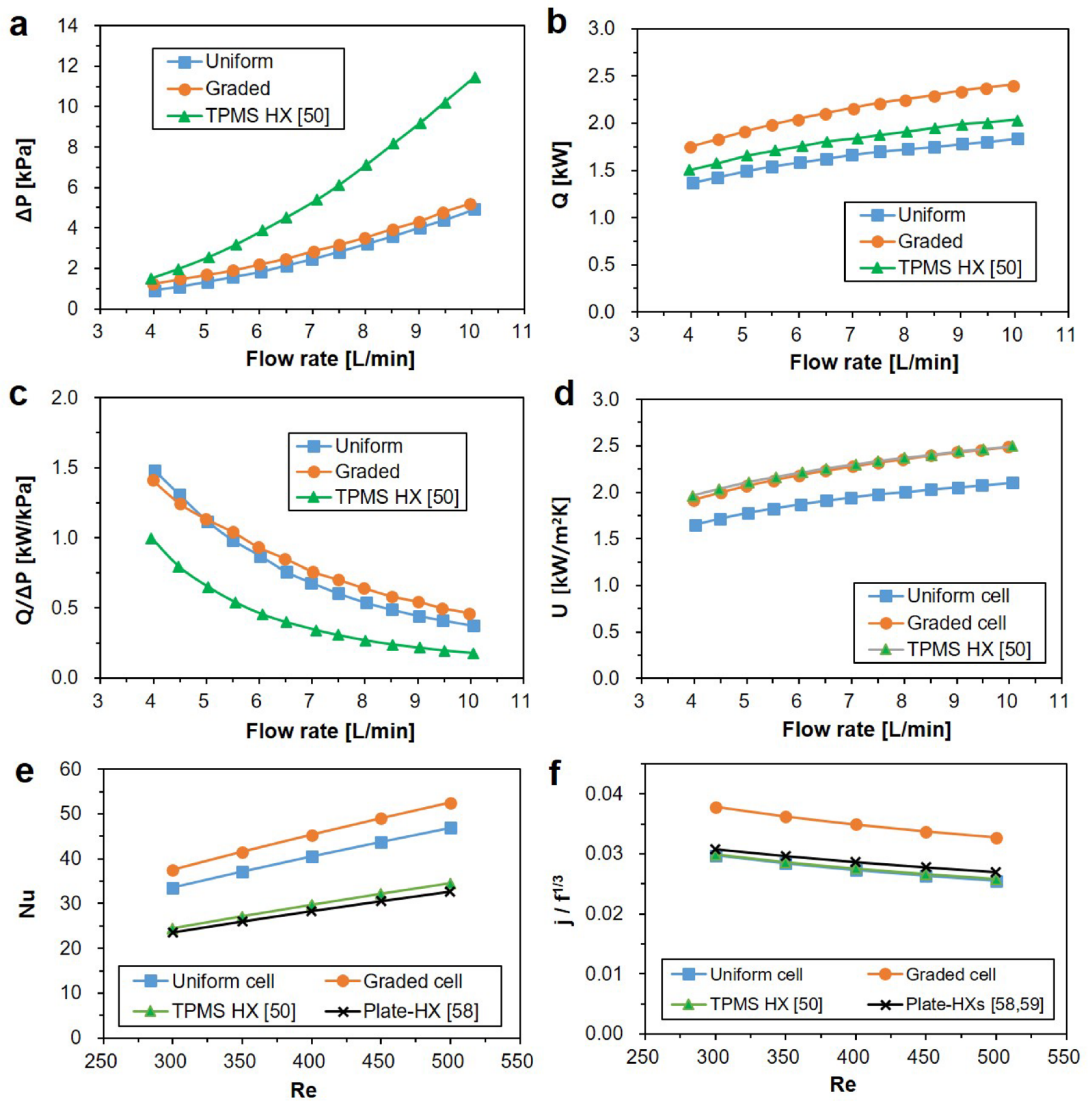
Comparison of experimental results: (**a**) pressure drop (Δ*P*), (**b**) heat exchange capacity (*Q*), (**c**) heat exchange capacity per unit pressure drop (*Q*/Δ*P*), (**d**) overall heat exchange coefficient (*U*), (**e**) Nusselt number (*Nu*), and (d) *j*/*f*^1/3^ ratio.

Experimental results are presented in Fig. 12, which also includes data from the authors’ previous study on TPMS HXs^50^ to examine the impact of graded cell sizing. Figure 12a compares the measured pressure drops between the uniform and graded TPMS HXs, revealing that both uniform and graded designs exhibit significantly lower pressure drops than those reported in the prior study. This substantial reduction in flow resistance is attributed to the proposed filtered gradation, where the tapered cylindrical domains effectively facilitate fluid entry into the TPMS channels.

Between these two designs, the graded cell configuration exhibits slightly higher pressure drops than the uniform cell design across all tested flow rates. The deviation between the two designs remains consistently around 0.3 kPa throughout the range of tested flow rates. For instance, at a flow rate of 6 L/min, the pressure drop deviation is 0.387 kPa, corresponding to a 21.3% increase compared to the uniform configuration. This increase is attributed to the reduction in hydraulic diameter in the graded design, as shown in Table 1, where the hydraulic diameter for the graded design (4.59 mm) represents a 22.8% reduction compared to the uniform design (5.95 mm). Despite the increase in flow resistance, the graded HX’s uniform flow distribution reduces dead zones, resulting in a slight mitigation of pressure drops.

Further investigation into the pressure drop differences (Δ*P*_graded_—Δ*P*_uniform_) and relative ratio (Δ*P*_graded_/Δ*P*_uniform_) between the uniform and graded designs was conducted by comparing the experimental results with CFD simulations. Detailed data are provided at supplementary Table S3. The experimental results show pressure drops of 1.811 kPa for the uniform design and 2.198 kPa for the graded design, which are higher than the simulation results of 1.004 kPa and 1.120 kPa, respectively.

These discrepancies arise from both simulation and experimental uncertainties. Although approximately 169 million elements were used in the simulations, this resolution might be insufficient to capture localized turbulent flows within the complex TPMS channels. In the experiments, the uncertainties in pressure drop measurements were calculated to be ± 8.54% for the uniform design and ± 7.03% for the graded design, as listed in Table 1. Additionally, the additively manufactured TPMS structures exhibited slightly increased wall thickness, 0.526 ± 0.04 mm for the uniform design and 0.533 ± 0.05 mm for the graded design, which reduced channel widths and contributed to increased pressure drops. Nevertheless, the relative pressure drops (Δ*P*_graded_/Δ*P*_uniform_) are similar between the simulation (1.205) and experimental results (1.213), indicating that the simulations provide a qualitatively reliable prediction of the pressure drop behavior between the two designs.

Figures 12b illustrate the variations in heat exchange capacity (*Q*) as the flow rate increases. The graded cell design demonstrates a significant improvement in heat exchange capacity, achieving approximately 30% higher value than the uniform design, and 20% higher than the previous TPMS-based HXs^50^. This remarkable enhancement can be attributed to the increased heat exchange area provided by the graded design, which facilitates more effective thermal interactions between the hot and cold fluids.

For a comprehensive evaluation of thermofluidic characteristics, the heat exchange capacity per unit pressure drop (*Q/ΔP*) is compared in Fig. 12c, demonstrating significant improvements in both the uniform and graded TPMS designs relative to the previous study^50^. At flow rates below 5 L/min, the *Q/ΔP* of the graded TPMS design is slightly lower than that of the uniform design. However, above this threshold, the graded design outperforms the uniform design, exhibiting a higher *Q/ΔP*. This trend reflects the trade-off between increased pressure drop and enhanced heat exchange capacity in the graded configuration. The flow rate of 5 L/min serves as a break-even point, where the advantages of improved thermal performance begin to outweigh the drawbacks of additional flow resistance.

Figure 12d presents the variation in the overall heat transfer coefficient (*U*) with increasing flow rate. For the uniform cell design, *U* ranges from 1651 to 2102 W/m^2^ K, while the graded design exhibits higher values, ranging from 1914 to 2488 W/m^2^ K. This enhancement is attributed to the balanced design of the graded TPMS, which optimally increases the surface area with only a minimal rise in flow resistance. The graded TPMS HX exhibits superior heat transfer performance compared to conventional water-to-water HXs, which typically have *U* values in the range of 850–1700 W/m^2^K^57^. These results highlight the superior thermal efficiency of the graded design and underscore its strong potential for high-performance HX applications.

To assess the overall performance of the uniform and graded TPMS HXs, key dimensionless quantities were compared with those from the authors’ previous TPMS-based HX design^50^ and with conventional plate-type HXs reported in previous studies^58,59^. The reference correlations for the Nusselt number (*Nu*), the Colburn *j*-factor (*j*), and the friction factor (*f*) were derived from experimental data on plate-type HXs with varying corrugated plate geometries and chevron angles. Therefore, these correlations are considered representative of the typical thermofluidic performance of conventional plate-type HXs.

Figure 12e presents the variation of *Nu* with respect to the Reynolds number (*Re*), demonstrating that both TPMS HX designs consistently outperform plate-type HXs^58^ and the previously developed TPMS HX^50^ across the entire range of *Re*. Since the Nusselt number, defined as *Nu* = *hD*_*h*_*/k*, reflects the convective heat transfer efficiency, higher *Nu* values indicate enhanced thermal performance. Consequently, the heat transfer rates for both TPMS HX designs exhibit superior heat transfer rates compared to plate-type HXs under various flow conditions. Furthermore, the graded TPMS design achieves *Nu* values that are 11.8% higher than those of the uniform design, highlighting the benefit of the graded configuration in enhancing convective heat transfer.

To comprehensively analyze overall performance considering both heat transfer and pressure drop characteristics, the *j*/*f*^1/3^ ratios of the uniform and graded TPMS HXs are compared with those of conventional plate-type HXs, as illustrated in Fig. 12f. The TPMS HXs with uniform cell configurations and the previously developed TPMS HX^50^ exhibit slightly lower *j*/*f*^1/3^ ratios than conventional plate-type HXs. In contrast, the graded TPMS HX demonstrates significantly higher performance, outperforming other HXs by approximately 28%. These findings highlight that the graded TPMS HX design achieves an optimal result balancing between heat transfer and flow resistance, leading to enhanced performance and positioning it a more efficient alternative to conventional HX designs.

## Conclusion

This study proposed a multifunctional gradation approach for the design of TPMS-based HXs, employing gradual modifications of the SDF to satisfy diverse functional requirements. The first gradation, referred to as filtering gradation, modified the morphology of the inlet and outlet regions to selectively guide the fluid flow, achieving a 50% reduction in flow resistance. The second gradation introduced a spatially varying cell size to improve flow uniformity, resulting in a 36% reduction in dead zones compared to the uniform cell design. To complement the cell-size gradation, a level-set gradation was applied to maintain a consistent wall thickness of 0.5 mm, thereby mitigating localized stress concentrations. The optimal TPMS HX design employed a cell size gradation from 6 to 10 mm and was additively manufactured with consistent wall thickness (0.533 ± 0.05 mm). Experimental evaluations demonstrated that the graded TPMS HX achieved a 30% enhancement in heat exchange capacity and a 28% improvement in lower *j*/*f*^1/3^ ratio compared to the uniform design, highlighting the efficacy of the proposed design methodology.

Because TPMS structures have inherently complex geometries, their manual modifications are highly challenging. Therefore, the proposed gradation approach based on the SDF manipulation provides a straightforward and efficient means of altering TPMS morphology. This method enables facile modification by allowing the superposition of multiple gradation functions tailored to predefined functional requirements. Compared to previous studies that focused solely on individual size or thickness gradation, the proposed multifunctional gradation scheme supports a more comprehensive and versatile design optimization process.

While this study focused on maintaining consistent wall thickness under adaptive cell-size gradation for HX applications, future work will explore wall thickness optimization based on structural FEAs, incorporating fluid–structure interaction (FSI) simulations. Furthermore, the gradation function itself can be further optimized to simultaneously maximize flow uniformity and heat transfer performance. Such multifunctional optimization of the graded TPMS design is expected to broaden its applicability across diverse industrial sectors.

## Electronic supplementary material

Below is the link to the electronic supplementary material.

Supplementary Material 1

## Data Availability

All data generated or analyzed during this study are included in this published article and its supplementary information files.
