# Supplementary material for: Multifunctional gradations of TPMS architected heat exchanger for enhancements in flow and heat exchange performances
Source: Sci Rep. 2025 Jun 6;15:19931. doi: 10.1038/s41598-025-04940-2 (PMC12144117; doi:10.1038/s41598-025-04940-2)
Supplement: Supplementary file 1 — Supplementary Material 1 [file 41598_2025_4940_MOESM1_ESM.docx]

Supplementary information on

**Multifunctional Gradations of TPMS Architected Heat Exchanger for Enhancements in Flow and Heat Exchange Performances**

Seo-Hyeon Oh, Jeong Eun Kim, Chan Hee Jang, Jungwoo Kim, Chang Yong Park,

and Keun Park

**Table S1.** Center positions of the taper-hole type filtering domains.

| *i* | Description | (*x_1_, y_1_, z_1_*) | (*x_2_, y_2_, z_2_*) |
| --- | --- | --- | --- |
| 1 | Hot inlet | (-40, 21, 22) | (-43, 24, -16) |
| 2 | Hot outlet | (40, -21, 22) | (43, -24, -16) |
| 3 | Cold inlet | (40, 21, 22) | (43, 24, -16) |
| 4 | Cold outlet | (-40, -21, 22) | (-43, -24, -16) |

**Table S2.** Comparison of accuracy of the additively manufactured TPMS HXs.

| Item | Uniform (*l* = 10 mm) | | Uniform (*l_min_* = 6 mm) | |
| --- | --- | --- | --- | --- |
|  | Designed | Measured | Designed | Measured |
| Length (mm) | 102.0 | 102.04 | 102.0 | 101.90 |
| Width (mm) | 64.0 | 63.93 | 64.0 | 63.81 |
| Height (mm) | 50.0 | 51.42 | 50.0 | 51.36 |
| Mass (g) | 340.7 | 347.9 | 357.1 | 360.8 |

**Table S3.** Comparison of pressure drops between the CFD simulation and experimental results (flow rate: 6 L/min).

| Method | Pressure drop (kPa) | | Δ*P_graded_ -* Δ*P_uniform_* | Δ*P_graded_ /* Δ*P_uniform_* |
| --- | --- | --- | --- | --- |
|  | Δ*P_uniform_* | Δ*P_graded_* | (kPa) |  |
| Simulation | 1.004 | 1.210 | 0.206 | 1.205 |
| Experiment | 1.811 | 2.198 | 0.387 | 1.213 |
